# Immunomodulatory Role of Kupffer Cell in Liver Allografts

**DOI:** 10.1186/1476-5926-2-S1-S32

**Published:** 2004-01-14

**Authors:** Zhaoli Sun, Tatehiko Wada, Sumito Hoshino, Keiichiro Uchikura, Andrew S Klein

**Affiliations:** 1 Department of Surgery, The Johns Hopkins University School of Medicine, Baltimore, MD 21205, USA

## Introduction

Liver transplantation is widely used as a treatment for end-stage liver disease. Liver allografts seem privileged compared with other solid organ grafts for a number of reasons. Successful liver transplantation can be performed across major histocompatibility complex (MHC) disparities, and the liver is the only solid organ that, when transplanted, can protect other co-transplanted syngeneic organs from immunological tissue destruction [1]. A proportion of patients with stable function can have immunosuppression reduced or withdrawn completely [2,3]. The reasons for this "liver effect" are not understood. Recently, there has been interest in lymphocyte apoptosis that occurs in OLT animal models. Allograft tolerance can be readily induced in experimental animals, particularly rodents, and spontaneous tolerance of mismatched liver grafts occurs in some train combinations [4]. In a spontaneously tolerant murine model of liver transplantation, the development of tolerance was associated with a higher rate of apoptosis of cells in the portal inflammatory infiltrate [5]. Sharland et al. [6], in a rat model, also showed that tolerant animals had higher numbers of apoptotic cells; they demonstrated by double staining that these were T lymphocytes.

Kupffer cells (KC), the resident macrophage population in the liver, are found within the sinusoidal lumen, adhering to the liver sinusoidal endothelial cells (LSEC). KC, which comprise one of major populations (20%) of the hepatic nonparenchymal cell fraction (NPC), can directly interact with passenger leukocytes and thus may play a role in immunomodulation and the induction of tolerance [7]. Following liver transplantation, donor KC not only migrate into the recipient lymph nodes, but also can be quickly replaced by recipient-derived monocytes [8,9]. Thus, KC are uniquely positioned for regulation of the T cell response in the liver. Our previous studies have shown that KC play an immunomodulatory role as manifested by inhibiting T lymphocyte proliferation in response to alloantigen stimulation. The purpose of this study was to quantify the effects of liver transplantation on KC immunomodulatory function, focusing specifically on Fas ligand (FasL) expression and their role in allo-reactive T cell apoptosis in chronic accepted and acutely rejected hepatic allografts.

## Methods

### Animals

Male Lewis (RT1^1^), and DA (RT1^ab^) rats were purchased from Harlan Sprague-Dawley (Indianapolis, IN) and used at 8–12 wk of age. Animals were maintained in the specific pathogen-free facility of Johns Hopkins Medical Institutions. Animals were cared for according to NIH guidelines and under a protocol approved by the Johns Hopkins University Animal Care Committee.

### Orthotopic liver transplantation

Orthotopic liver transplantation (OLT) was performed under methoxyflurane (Medical Development, Springvale, Australia) inhalation anesthesia, according to a method modified from that described by Kamada and Calne [10]. The hepatic artery was not reconstructed. Three combinations were selected: 1) a model of chronic allograft acceptance (Lewis into DA, **OLT**_R_); 2) a model of acute allograft rejection (DA into Lewis, **OLT**_A_); and 3) a model of syngeneic OLT (Lewis into Lewis, **OLT**_S_). Allograft survival was determined by recipient survival and rejection was confirmed histologically.

### Lymphocyte preparation and Allo-reactive T cells activation

Suspensions of spleen cells (SC) were prepared by passage of mechanically disunited spleens through a 50 micrometer stainless steel screen, erythrocyte lysis with tris-ammonium chloride, and three washings in RPMI 1640 (1200 rpm for 5 min). From these SC suspension, T-lymphocytes and APCs were further purified by sequential passage over nylon wool columns. Allo-reactive T cells (**ATC**) were generated by incubation of either Lewis T cells with –-irradiated DA-APCs (2200 rads) or DA T cells with irradiated Lewis-APCs for 5 days.

### KC isolation

KC isolation was performed by 0.05% collagenase perfusion of the liver, isopycnic sedimentation in two-step Percoll gradient (25% and 50% Percoll), and selective adherence of the cell to plastic flasks [11,12]. This technique of cell isolation yielded, on average, 40 to 60 million KC per liver, with 90–95% viability as determined by trypan blue exclusion. The cells showed typical macrophage morphologic features and stained positively for nonspecific esterase and both ED1 and ED2, and phagocytosed 1 micrometer Latex beads. In addition, these cells express the Kupffer cell receptor (KCR) confirmed using RT-PCR. Purity of the KC fraction was consistently >95%.

### Quantitative RT-PCR for Fas ligand mRNA expression

To assess FasL mRNA expression, total RNA was subjected to RT-PCR using the following primers: 5'-ATGCAGCAGCCCATG-3' and 5'-AAGCTTATACAAGCCGAA-3'. Primers for beta-actin were purchased from Clontech (Palo Alto, CA).

### Western blots

KC proteins were extracted and whole cell extracts were subjected to 12% SDS-PAGE. Resolved proteins were transferred to a nitrocellulose membrane and incubated with anti-FasL antibody (clone 33, BD PharMingen), and followed with HRP-conjugated secondary antibody (1:1000). After three 10-min washes with PBS-Tween 20, Peroxidase activity was visualized with the ECL kit (Amersham Pharmacia Biotech, Piscataway, NJ) according to the manufacturer's instruction.

### Cytotoxicity of KCs against allo-reactive T cells (ATC)

ATC were cultured with ^3^H-thymidine (5 –Ci/ml) for 12 hours. ^3^H-thymidine labeled ATCs (1 – 10^5 ^cells/well) and KC were co-cultured in 96 well plate at different E:T ratios starting at 1:1 in 200 microliters of complete medium for 4 hours. Cells were harvested and thymidine release was calculated as follows: % thymidine release = [cpm (without effectors)-cpm (with effectors) / cpm (without effectors)] – 100.

### Inhibitory effect of anti-FasL antibody on cytotoxicity of KCs

To test the role of FasL in the cytotoxicity of KCs against ATC, we measured 3H-thymidine release in a KC and ATC co-culture system pulsed with the anti-FasL antibody or a control IgG (BD PharMingen) at antibodies concentration 0.3 – 10 micrograms/ml.

## Results

**Figure 1:**
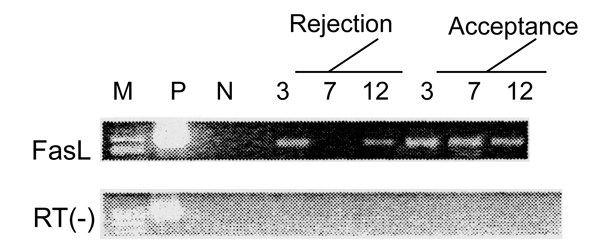
FasL mRNA expression of KC post-OLT determined by RT-PCR.

**Figure 2:**
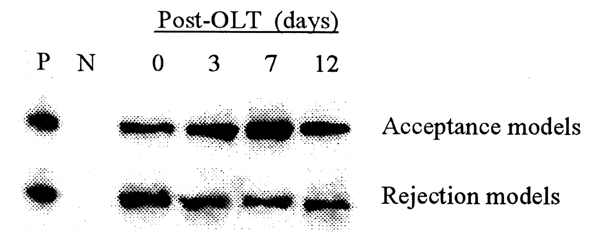
FasL protein in KC post-OLT quantified using Western blot.

KC express FasL. In acceptance and rejection liver transplant models, we measured KC FasL mRNA and protein using RT-PCR and Western blotting, respectively. On post transplantation days 3, 7 and 12, FasL mRNA expression of KC recovered from the acceptance model was significantly higher than that measured from KC recovered from rejecting allografts (Fig. 1). FasL protein was also higher in the acceptance model than that in rejection model, with peak levels noted on day 7 following liver transplantation (Fig. 2).

**Figure 3:**
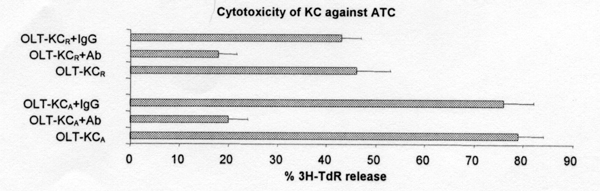
Cytotoxicity of KC against ATC post OLT. KC recovered from liver allografts on day 7 post-OLT. OLT-KC_A_: KC recovered from acceptance models; OLT-KC_R_: KC recovered from rejected models. KC/AT_C _ratio = 10/1. Ab: anti-FasL antibody; IgG: mouse IgG as control antibody. Antibody concentration: 10 micrograms/ml.

Cytotoxicity of KC against ATC dependent on FasL expression. Since activated T cells express elevated level of Fas, we then determined the cytotoxicity of KC against alloreactive T cells (ATC). Coculture of ATC with non-transplanted Lewis or DA KC resulted in increased T cell lysis compared to controls. KC recovered from either transplant model had an increased ability to lyse ATC compared with non-transplanted controls. Furthermore, KC recovered from the acceptance model had a greater ability to lyse ATC than KC from the rejection model seven days post-transplantation. However, this lysis was significantly suppressed by the addition of neutralizing anti-FasL antibody (Fig. 3).

## Discussion

We have demonstrated that KC induced apoptosis and lysis of allo-reactive T cells, and this lysis effect was inhibited by anti-FasL antibody. KC FasL expression is increased following liver transplantation and associated with increased ability of KC lyses ATC. These observations are consistent with the hypothesis that KC act as FasL-expressing APCs, and may have the ability to induce and maintain immune tolerance. Our results in this study give rise to supplementary evidence that KC induce activated T cells apoptosis; thus KC may participate in the development of immune tolerance following allogeneic liver transplantation.

Apoptosis of T cells mediated by FasL in a paracrine fashion has been shown previously to be critical for the maintenance of the immunoprivileged site [13,14]. A recent study suggests that the maintenance of immunoprivilege involves the induction of systemic T cell tolerance [15]. Induction of Ag-specific T cell tolerance by FasL-transfected APCs suggests a novel strategy for modulating the T cell response [16]. Regulation of FasL expression in KC or macrophages has been achieved in a number of experiments. IFN-gamma induced overexpression of FasL in KC has been proposed as a mechanism of hepatic immunoregulation [17]. Replacement of donor KC by recipient-derived KC following OLT may also influence the FasL expression of KC in liver allografts. For example, FasL expression is higher in DA rat KC than in Lewis rat KC. Donor Lewis hepatic KC replacement by recipient DA KC following liver transplantation results in increased FasL expression by KC in the liver allografts. Whether a genetic difference in KC FasL expression contributes to allograft acceptance in this model remains to be elucidated.

In conclusion, our findings identify an immunoregulatory mechanism by which KC induce T cell apoptosis via Fas/FasL pathway. This immunoregulatory ability of KC is dramatically increased following liver transplantation, especially in KC recovered from chronically accepted liver allografts. These findings support our hypothesis that KC-dependent T cell deletion via the Fas/FasL pathway may play an important role in the induction of immune tolerance following liver transplantation.
